# Peer Review of “Use of Spinal Anesthesia in Pediatric Laparoscopic Appendectomies: Case Series”

**DOI:** 10.2196/29604

**Published:** 2021-04-28

**Keywords:** pediatrics, appendectomy, spinal anesthesia, general anesthesia, laparoscopy, vomiting, keyhole, surgery, anesthesia, appendix

*This is a peer-review report submitted for the paper "Use of Spinal Anesthesia in Pediatric Laparoscopic Appendectomies: Case Series"*

## Round 1 Review

### General Comments

This paper [1] is a retrospective case-control comparative study between spinal and general anesthesia for laparoscopic appendectomy in children. The manuscript is very well written with a good study design and robust statistical analysis and display to defend the conclusion.

### Specific Comments

#### Minor Comments

1. Objective: “The objective of this study is to demonstrate that laparoscopic appendectomies are successful under spinal anesthesia and elicit clear advantages over general anesthesia.” The authors state the objectives with the conclusion in mind. Normally, the objective should be: “Comparing spinal and general anesthesia for the following variables…” This was done again in the last paragraph of the *Introduction* section where the final conclusion is stated.
2. Methods: Should include only what was done and how it was done; results (Table 1) should be moved to the *Results* section.
3. Results: In Table 2, it is not enough to have only a summary of the results (significantly higher or lower); the actual numbers should be presented.
4. Results: Should include only data and numbers with no discussion or comments (make sense, encouraging, etc).
5. Discussion: Please explain what is meant by extremity surgery here: “Laparoscopic surgery is now the method of choice for lower abdominal and (extremity) procedures.”

## Round 2 Review

### General Comments

The authors have adequately answered the reviewer’s comments.
